# Co-fermentation of *Propionibacterium freudenreichii* and *Lactobacillus brevis* in Wheat Bran for *in situ* Production of Vitamin B12

**DOI:** 10.3389/fmicb.2019.01541

**Published:** 2019-07-05

**Authors:** Chong Xie, Rossana Coda, Bhawani Chamlagain, Pekka Varmanen, Vieno Piironen, Kati Katina

**Affiliations:** Department of Food and Nutrition, University of Helsinki, Helsinki, Finland

**Keywords:** *Propionibacterium freudenreichii*, *Lactobacillus brevis*, bioreactor, vitamin B12, wheat bran, co-fermentation

## Abstract

The present study investigated the effect of co-fermentation on vitamin B12 content and microbiological composition of wheat bran. *Propionibacterium freudenreichii* DSM 20271 was used as the producer of vitamin while *Lactobacillus brevis* ATCC 14869 was selected to ensure the microbial safety of the bran dough. Fermentation trials were conducted in bioreactors to monitor and adjust the pH of the ferments. Vitamin B12 level reached 357 ± 8 ng/g dry weight (dw) after 1 day of pH-controlled fermentation with *P. freudenreichii* monoculture and remained stable thereafter. In co-fermentation with *L. brevis*, slightly less vitamin B12 (255 ± 31 ng/g dw) was produced in 1 day and an effective inhibition of the growth of total *Enterobacteriaceae* and *Bacillus cereus* was obtained. On day 3, vitamin B12 content in pH-controlled co-fermentation increased to 332 ± 44 ng/g dw. On the other hand, without a pH control, co-fermentation resulted in a stronger inhibition of *Enterobacteriaceae* and *B. cereus* but a lower level of vitamin B12 (183 ± 5 ng/g dw on day 3). These results demonstrated that wheat bran fermented by *P. freudenreichii* and *L. brevis* can be a promising way to produce vitamin B12 fortified plant-origin food ingredients, which could reduce cereal waste streams and contribute to a more resilient food chain.

## Introduction

Vitamin B12 plays an important role in human body and its deficiency may result in megaloblastic anemia, peripheral arterial diseases and various neurological disorders (Nielsen et al., 2012; Zsori et al., 2013). Previously, deficiency of this vitamin was considered as rare, but recent studies found that varying degrees of suboptimal vitamin B12 status, ranging from insufficiency to outright deficiency, have wide prevalence and affect people of all ages (Green et al., 2017; Smith et al., 2018). Considering animal products are the main dietary source of vitamin B12, developing plant-origin food products fortified with vitamin B12 is a promising way to increase dietary vitamin B12 intake of people consuming limited amounts of animal products (Watanabe et al., 2014). Among the plant-based food matrices, cereal and cereal bran are the most abundant in the world and are an excellent material for innovative food applications. As the by-product of wheat (*Triticum aestivum*) milling process, a huge quantity of wheat bran is produced every year and yet most of it is discarded or used for feed due to its poor technological performance (Coda et al., 2015). *Propionibacterium freudenreichii* is a generally recognized as safe (GRAS) bacterium with the ability to produce active vitamin B12 in different plant-based matrices (Chamlagain et al., 2017; Signorini et al., 2018; Wolkers-Rooijackers et al., 2018). In our previous work, we demonstrated the possibility of producing physiologically significant amount of active vitamin B12 in non-sterile wheat bran using fermentation with *P. freudenreichii* (Xie et al., 2018). However, growth of potential pathogens, such as enterobacteria, from endogenous microbiota during wheat bran fermentation may result in safety concerns of the fermented dough to be used in food applications (Xie et al., 2018).

Co-fermentation with lactic acid bacteria (LAB) could be a feasible solution to improve the microbiological safety of the fermented bran matrix. LAB are a group of bacteria widely used in cereal fermentation to improve the flavor, nutrient contents and texture of products (De Vuyst and Neysens, 2005). Moreover, LAB can also produce various natural antimicrobials, contributing to the safety of fermented food products (Leroy and De Vuyst, 2004; Axel et al., 2017; Leyva Salas et al., 2017). Cultivation of propionibacteria (PAB) with LAB in cheese production is a typical example of commensalism because lactic acid produced by LAB is the preferential carbon source for PAB (Smid and Lacroix, 2013). Co-cultivation of PAB and LAB is also an appropriate choice for industrial biopreservation due to their production of various antimicrobial compounds (Smid and Lacroix, 2013). In addition, a co-fermentation process of LAB–PAB has been shown to produce vitamin B12 and folate in sterilized whey permeate medium (Hugenschmidt et al., 2011). However, producing vitamin B12 during the co-fermentation of LAB–PAB in non-sterile wheat bran matrices has not yet been reported.

There is only a limited number of studies on co-fermentation of LAB-PAB in cereal-based products, and most of them are focused on the preservative effect of these cultures. For instance, a mixed culture pre-fermentation of LAB and PAB can improve the shelf-life of wheat or rye sourdough breads as a result of the acid production (Javanainen and Linko, 1993a,b). Tinzl-Malang et al. (2015) also reported the antifungal, texture-building and anti-staling ability of LAB-PAB co-fermentation in wheat bread due to the synergistic effects of exopolysaccharide and acid productions. Notably, pH control was used in all above mentioned studies, most probably to avoid inhibition of PAB growth and metabolism by rapid pH drop caused by acid producing LAB (Chaia et al., 1994).

The aim of this study was to investigate the production of vitamin B12 by *P. freudenreichii* DSM 20271 in wheat bran during its co-fermentation with *Lactobacillus brevis* ATCC 14869 with or without a pH control. The strain of *L. brevis* was chosen based on a pre-screening study to improve the microbial safety of the fermented bran. The acidification properties, microbial growth, sugar metabolism and the change in riboflavin (a precursor for synthesis of vitamin B12) content were also monitored to follow the microbial metabolism during the co-fermentation.

## Materials and Methods

### Pre-screening of Culture Combinations

To select a suitable culture in co-fermentation with *P. freudenreichii* for improving the microbial safety of fermented bran, *Saccharomyces cerevisiae* H10 and 7 strains of LAB belonging to the species: *Lactobacillus reuteri*, *Leuconostoc pseudomesenteroides*, *Lactobacillus delbrueckii*, *Weissella confusa*, *Leuconostoc mesenteroides*, and *L. brevis* (strain codes and origins in Supplementary Table S1), previously used for cereal or bran fermentation, were separately used for co-fermentation with *P. freudenreichii* DSM 20271. Wheat bran doughs (400 g) were prepared by mixing 80 g bran with 320 g water. After transferring into 500 ml bottles, doughs were inoculated at the initial cell density of 9.0 log colony forming units (CFU)/g of *P. freudenreichii* and 6.0 log CFU/g of LAB or yeast. The initial inoculum level of *P. freudenreichii* was performed according to our previous study to produce sufficient content of vitamin B12 (Xie et al., 2018). The inoculum levels of LAB or yeast were determined by preliminary experiments to achieve a significant inhibition on *Enterobacteriaceae* growth with a minor inhibition on production of vitamin B12. Doughs were fermented in shaking conditions (200 rpm) for 3 days at 25°C and during fermentation, pH value was measured every 12 h. When the value dropped below 5.5, pH was adjusted to 6.0 with 3M NaOH (no adjustment at 60 h). The fermentations were carried out in biological duplicate. After day 0, 1, and 3, samples of 20 g were taken out for cell count measurement of PAB and total *Enterobacteriaceae*. Based on the acidification properties (Supplementary Table S2) and the inhibitory effect on the propagation of *Enterobacteriaceae* (Supplementary Table S3), *L. brevis* ATCC 14869 was selected for further co-fermentation experiments.

### Raw Material, Microbial Strains, and Culture Preparation

The milled wheat bran was obtained from Fazer Mills (Lahti, Finland). More than 99% of bran particles are smaller than 790 μm and about 80% of them were larger than 224 μm. The composition of the bran was 16.0% protein, 20.0% available carbohydrate, 43.0% fiber, 4.8% lipids, 12.5% moisture, and 3.9% ash, as provided by the manufacturer.

Both *P. freudenreichii* and *L. brevis* cultures were cryopreserved at −60°C in glycerol. *P. freudenreichii* was propagated in the yeast extract lactate (YEL) medium (Malik et al., 1968) at 30°C for 3 days and *L. brevis* was propagated in de Man, Rogosa and Sharpe (MRS) medium (Lab M, Lancashire, United Kingdom) at 37°C for 1 day. After incubation, the cultures were recovered by centrifugation (3,200 × g, 10 min) and resuspended in MillQ water before inoculation.

### Fermentation

Four different wheat bran doughs were fermented as outlined in Table 1: spontaneously fermented bran dough with pH control (Control); bran dough fermented with *P. freudenreichii* monoculture with pH control (PF_C); bran dough fermented with *P. freudenreichii*/*L. brevis* co-culture with pH control (CO_C); bran dough fermented with *P. freudenreichii*/*L. brevis* co-culture without pH control (CO_U). In each fermentation, 1 kg of wheat bran dough was prepared by mixing bran and water in a 15:85 ratio. Wheat bran doughs were transferred in three bioreactors (Sartorius, Goettingen, Germany) and successively inoculated with the microbial starters. Fermentation was carried out for 72 h at 25°C, with stirring set at 600 rpm. In doughs fermented with pH control, 5 M NaOH solution was used to maintain the pH value at 5.0.

**TABLE 1:** PH and NaOH consumption (ml) during controlled fermentation.

**Sample code**	Starter^*^	**Initial pH**	**Final pH**	**Time (h)^∗∗^**	**NaOH consumption (5 M) after 1 day**	**NaOH consumption (5 M) after 3 day**
Control	–	6.5	5.0	19	11 ± 1^a^	32 ± 2^a^
PF_C	*P. f*	6.5	5.0	20	14 ± 1^b^	36 ± 2^a^
CO_C	*P. f + L. b*	6.5	5.0	11	24 ± 4^c^	43 ± 1^b^
CO_U	*P. f + L. b*	6.5	3.7	11	–	–

Control, spontaneously fermented bran with pH control; PF_C, bran fermented with *P. freudenreichii* and pH control; CO_C, bran fermented with *P. freudenreichii* and *L. brevis* with pH control; CO_U, bran fermented with *P. freudenreichii* and *L. brevis* without pH control. ^*^P. f, *P. freudenreichii*; *L. b*, *L. brevis*. ^∗∗^The time required for pH decreasing to 5.0. Values bearing different superscripts (a–c) in the same column are significantly different (*p* < 0.05).

*Propionibacterium freudenreichii* was inoculated at the initial cell density of 9.0 log colony forming units (CFU)/g and *L. brevis* at the level of 6.0 log CFU/g. At time 0, 24, and 72 h, samples of 80 g were taken. An aliquot of 10 g was used for the cell count determinations and the rest of the samples were stored (−20°C) for other analyses. Three biological replicate fermentations were carried out for each dough type.

### Microbial Counts

To estimate the number of viable cells, bran doughs (10 g) were serially diluted in sterile saline solution (8.5 g/L of NaCl) and appropriate dilutions were plated on the agar plates. YEL plates were incubated anaerobically for 4 days in anaerobic jars with Anaerogen (Oxoid, Basingstoke, United Kingdom) followed by 1 day incubation under aerobic conditions at 30°C. In these conditions, the colonies of *P. freudenreichii* turn brownish to be distinguishable from colonies of other bacteria. MRS agar (Lab M) for the cell counts of LAB was supplemented with 0.01% of cycloheximide (Sigma Chemical Co., United States) and microaerobically incubated at 30°C for 48 h. Plate count agar (PCA) plates (Lab M) were used for the cell counts of total aerobic bacteria. Yeast and mould (YM) agar plates (3 g/L malt extract, 3 g/L yeast extract, 5 g/L peptone, 10 g/L dextrose, and 0.01% chloramphenicol) were used for the cell counts of yeast. Total *Enterobacteriaceae* were enumerated on the violet red bile glucose agar (VRBGA) plates (Lab M). Polymyxin egg yolk mannitol bromothymol blue agar (PEMBA) plates (Lab M) were used for the cell counts of *Bacillus cereus*. PCA, YM, and PEMBA plates were incubated aerobically at 30°C for 48 h, VRBGA plates were incubated aerobically at 37°C for 48 h.

### Determination of Acids

Lactic acid, acetic acid and propionic acid were determined using a high-performance liquid chromatography (HPLC) method. Dough samples (1 g) after dilution (1:10, w/v) in MilliQ water were centrifuged (3,200 × g, 10 min) and supernatants were filtered (0.45 μm, Pall, United States) before injection. HPLC analysis was performed with the same instrument and the method as reported in the earlier study (Xie et al., 2018).

### Determination of Monosaccharides

Arabinose, galactose, xylose, glucose, and fructose were analyzed by high performance anion exchange chromatography equipped with a pulse amperometric detection system (HPAEC-PAD). Before analysis, dough samples diluted in water (1:10, w/v) were filtered by an Amicon Ultra-0.5 centrifugal filter unit (Millipore, Billerica, MA, United States) at 12,000 × g for 10 min to get rid of polymeric molecules. Monosaccharides were separated on a CarboPac PA1 column (250 × 4 mm i.d., Dionex, Sunnyvale, CA, United States) and detected using a Waters 2465 pulsed amperometric detector (Waters, United States). The solvents used were 200 mM NaOH and MilliQ water. A gradient elution was maintained at a constant flow rate of 1 ml/min: 0–31 min, 2 mM NaOH; 31–33 min, 200 mM NaOH; and 33–50 min, 2 mM NaOH, with an additional 10 min washing and regeneration steps. The injection volume was 10 μl. Glucose (Merck, Germany), fructose (Merck), xylose (Merck), arabinose (Merck), and galactose (Merck) were used as external standards and 2-deoxy-D-galactose (Sigma-Aldrich, Germany) was used as the internal standard for quantification.

### Determination of Vitamin B12

Vitamin B12 in the bran dough was extracted in cyano form and determined by an Ultra-HPLC (UHPLC) method as described by Xie et al. (2018). During determination, the presence of other corrinoids, especially pseudovitamin B12, was followed in the chromatograms based on their retention times and absorption spectra according to our previous studies (Chamlagain et al., 2015, 2017; Deptula et al., 2015).

Briefly, dough samples (3 g) were mixed with 15 ml of extraction buffer (8.3 mM sodium hydroxide and 20.7 mM acetic acid, pH 4.5) and 100 μl of sodium cyanide (1% w/v in water). After extraction in boiling water (30 min), cooled mixtures were incubated in a water bath (30 min, 37°C) with addition of 300 μl α-amylase (50 mg/ml; St Louis, MO, United States) to allow the breakdown of starch before centrifugation (6,900 × *g*, 10 min). Residues after centrifugation were suspended in 5 ml of extraction buffer and centrifuged again. Both supernatants were combined and adjusted to the same volume (25 ml) with the extraction buffer. Finally, 10 ml of the extracts were purified using an immunoaffinity column (Easi-Extract; R-Biopharm; Glasgow, Scotland) and analyzed with a Waters UPLC system (Milford, MA, United States) as explained by Chamlagain et al. (2015).

### Determination of Riboflavin

Content of riboflavin in doughs was determined with a UHPLC method according to Chamlagain et al. (2016) with minor modification. Samples (2 g) were mixed with 15 ml of 0.1 M hydrochloric acid and extracted in a boiling water bath (60 min). After cooling on ice, the pH of the mixture was adjusted to 4.5 with 2.5 M sodium acetate and incubated at 37°C with Taka-Diastase (50 mg; Pfaltz and Bauer, CT, United States) and β-amylase (5 mg; Sigma-Aldrich) for 24 h. The extract was filtered (0.2 μm, Pall, United States) and analyzed on a Waters UPLC system with an Acquity BEH C18 column (2.1 × 100 mm, 1.7 μm) and a Waters fluorescence detector using aqueous methanol (30% v/v) containing 20 mM ammonium acetate as an eluent (0.2 ml/min).

### Statistical Analysis

Statistical analysis was performed using SPSS 24.0 for Windows (IBM Corporation, NY, United States). One-way analysis of variance (ANOVA) and Tukey’s *post hoc* test were used to determine significant differences at a *p*-value < 0.05 among the samples.

## Results

### Pre-screening of Co-fermentation Cultures

Supplementary Tables S2–S4 show the change in pH values and the cell counts of total *Enterobacteriaceae* and PAB during bran dough fermentation with *P. freudenreichii* monoculture and in co-fermentation of *P. freudenreichii* with LAB/yeast strains. In general, dough pH dropped more rapidly and lower cell densities of *Enterobacteriaceae* were counted in doughs co-fermented with LAB compared to doughs co-fermented with yeast or fermented with *P. freudenreichii* monoculture. Fastest drop in pH and the lowest cell counts of *Enterobacteriaceae* on both day 1 (2.4 ± 0.2 log CFU/g) and day 3 (3.2 ± 0.2 log CFU/g) were observed in fermentations including *L. brevis* as a starter. The cell density of *P. freudenreichii* in all combinations varied from 8.9 to 9.4 log CFU/g during fermentation.

### Microbial Counts of Bran Doughs

In the control dough, no PAB were detected throughout the fermentation (Table 2). The initial cell density of *P. freudenreichii* was ca. 8.7 log CFU/g on day 0 due to the inoculum. In the PF_C and CO_C doughs, cell density of *P. freudenreichii* increased from ca. 8.7 log CFU/g to ca. 9.2 log CFU/g during the first day and remained stable thereafter. In the CO_U dough, the cell density of *P. freudenreichii* remained constant from day 0 to day 3.

**TABLE 2:** Cell counts (log CFU/g) of *Propionibacteria* (PAB), lactic acid bacteria (LAB), total aerobic bacteria, yeasts, total *Enterobacteriaceae*, and *Bacillus cereus* during bran dough fermentation.

**Time (days)**	**0**	**1**	**3**
**PAB**			
Control	nd^*^	nd	nd
PF_C	8.8 ± 0.1^a,x^	9.2 ± 0.1^b,y^	9.0 ± 0.1^b,y^
CO_C	8.7 ± 0.1^a,x^	9.1 ± 0.1^b,y^	8.9 ± 0.2^b,xy^
CO_U	8.6 ± 0.1^a,x^	8.6 ± 0.2^a,x^	8.5 ± 0.1^a,x^
**LAB**			
Control	2.7 ± 0.3^a,x^	9.8 ± 0.2^a,y^	9.8 ± 0.2^a,y^
PF_C	3.0 ± 0.2^a,x^	9.7 ± 0.2^a,y^	9.6 ± 0.1^a,y^
CO_C	6.3 ± 0.2^b,x^	10.2 ± 0.0^b,y^	10.3 ± 0.2^b,y^
CO_U	6.4 ± 0.2^b,x^	9.6 ± 0.2^a,z^	9.1 ± 0.1^a,y^
**Total aerobic bacteria**			
Control	5.2 ± 0.2^a,x^	9.8 ± 0.4^a,y^	9.8 ± 0.1^b,y^
PF_C	5.2 ± 0.0^a,x^	9.6 ± 0.2^a,y^	9.6 ± 0.2^b,y^
CO_C	6.4 ± 0.3^b,x^	9.9 ± 0.3^a,y^	10.2 ± 0.2^c,y^
CO_U	6.5 ± 0.2^b,x^	9.7 ± 0.2^a,z^	9.2 ± 0.2^a,y^
**Yeasts**			
Control	3.7 ± 0.3^a,x^	5.1 ± 0.0^b,y^	5.4 ± 0.1^b,y^
PF_C	3.7 ± 0.1^a,x^	5.1 ± 0.2^b,y^	5.3 ± 0.1^b,y^
CO_C	3.6 ± 0.2^a,x^	3.6 ± 0.2^a,x^	5.1 ± 0.3^b,y^
CO_U	3.6 ± 0.1^a,x^	3.4 ± 0.2^a,x^	3.6 ± 0.2^a,x^
**Total *Enterobacteriaceae***			
Control	4.8 ± 0.0^a,y^	6.1 ± 0.1^b,z^	3.7 ± 0.3^b,x^
PF_C	4.7 ± 0.1^a,y^	6.0 ± 0.1^b,z^	3.7 ± 0.1^b,x^
CO_C	4.7 ± 0.1^a,y^	3.3 ± 0.3^a,x^	3.4 ± 0.4^b,x^
CO_U	4.8 ± 0.1^a,z^	3.4 ± 0.3^a,y^	2.8 ± 0.1^a,x^
***Bacillus cereus***			
Control	3.2 ± 0.1^a,x^	3.1 ± 0.0^b,x^	nd
PF_C	3.3 ± 0.0^a,x^	3.4 ± 0.1^b,x^	nd
CO_C	3.2 ± 0.2^a,y^	2.4 ± 0.1^a,x^	nd
CO_U	3.1 ± 0.1^a^	nd	nd

^*^nd, not detected. Control, spontaneously fermented bran with pH control; PF_C, bran fermented with *P. freudenreichii* and pH control; CO_C, bran fermented with *P. freudenreichii* and *L. brevis* with pH control; CO_U, bran fermented with *P. freudenreichii* and *L. brevis* without pH control. The results are expressed as the mean ± standard deviation (*n* = 3). Values from the same day and microbial group bearing different superscripts (a–c) are significantly different (*p* < 0.05). Values from the same dough type and microbial group bearing different superscripts (x–z) are significantly different (*p* < 0.05).

In doughs without *L. brevis* inoculation (control and PF_C), the initial cell density of LAB was ca. 3.0 log CFU/g and increased to ca. 9.8 log CFU/g on day 1. In the CO_C and CO_U doughs, the initial cell densities of LAB were ca. 6.3 log CFU/g and increased to ca. 10.2 log CFU/g and ca. 9.6 log CFU/g on day 1, respectively. From day 1 to day 3, cell density of LAB remained stable in the CO_C dough but decreased of 0.5 log units in the CO_U dough. The initial cell densities of the total aerobic bacteria ranged from ca. 5.2 log CFU/g to 6.4 log CFU/g. During fermentation, their cell densities increased in the range of ca. 9.2 log CFU/g to 10.2 log CFU/g.

The initial cell density of yeasts was approximately 3.7 log CFU/g on day 0. The cell density of yeasts increased to ca. 5.1 log CFU/g on day 1 in doughs without *L. brevis* inoculation but remained unaltered afterward in co-fermented doughs. On day 3, yeast cell number in the CO_U dough was significantly (*p* < 0.05) lower than in the other doughs.

Before fermentation, ca. 4.8 log CFU/g of total *Enterobacteriaceae* and ca. 3.2 log CFU/g of *B. cereus* were found in wheat bran dough. In the control and PF_C doughs, cell densities of *Enterobacteriaceae* increased to ca. 6.0 log CFU/g on day 1 and decreased to ca 3.7 log CFU/g on day 3. In the CO_C dough, cell density of total *Enterobacteriaceae* was ca. 3.3 log CFU/g on day 3. In the CO_U dough, the final cell density of *Enterobacteriaceae* (2.8 ± 0.1 log CFU/g) was significantly (*p* < 0.05) lower than in the other doughs. *B. cereus* was not detected in the CO_U dough but a cell density of 2.4 to 3.0 log CFU/g of *B. cereus* was still detected in the other 3 doughs after 1 day of fermentation. However, on day 3, *B. cereus* was not found in any of the four doughs.

### Acidification of the Doughs

On day 0, the pH value of all the doughs were ca. 6.5 (Table 1). In the doughs inoculated with *P. freudenreichii*/*L. brevis* co-culture pH dropped most rapidly and reached pH 5.0 already after 11 h. In the dough inoculated with *P. freudenreichii* monoculture and in the control dough, pH dropped similarly and reached pH 5.0 after 20 and 19 h, respectively. At the end of fermentation, pH 3.7 was reached in the CO_U dough while in other doughs pH remained at 5.0.

Among the doughs with pH control, the highest consumption of NaOH solution (5 M) was found in the CO_C dough both on day 1 (24 ± 4 ml) and day 3 (43 ± 1 ml). The consumption of NaOH in the PF_C dough (14 ± 1 ml) was significantly (*p* < 0.05) higher than in the control dough (11 ± 1 ml) on day 1 but there was no significant (*p* > 0.05) difference in NaOH consumption between these two doughs on day 3.

**FIGURE 1:**
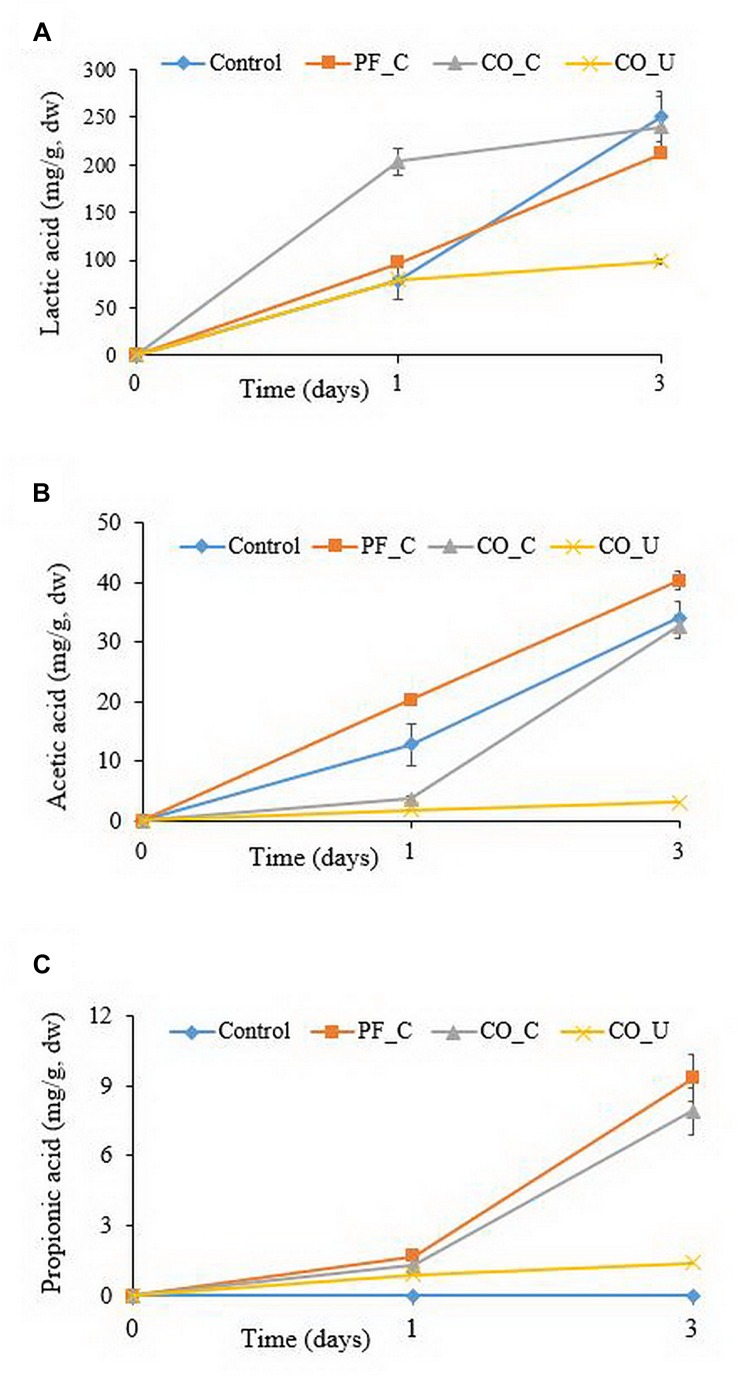
Concentration (mg/g, dry weight) of lactic acid **(A)**, acetic acid **(B)**, and propionic acid **(C)** during fermentation. Values are means and standard deviations of three replicates. Control, spontaneously fermented bran with pH control; PF_C, bran fermented with *P. freudenreichii* monoculture and pH control; CO_C, bran fermented with *P. freudenreichii/L. brevis* co-culture with pH control; CO_U, bran fermented with *P. freudenreichii/L. brevis* co-culture without pH control.

Before fermentation, lactic, acetic and propionic acid were not detected in any of the doughs (Figure 1). On day 1, the concentration of lactic acid in the CO_C dough was 204 ± 14 mg/g dry weight (dw), which was significantly (*p* < 0.05) higher than in the other doughs (ranging from ca. 79 to 96 mg/g dw). On day 3, lactic acid content in the doughs with a pH control had no significant (*p* > 0.05) difference and varied from 212 to 250 mg/g dw. In the CO_U dough, the concentration of lactic acid was 98 ± 2 mg/g dw at day 3.

On day 1, the highest amount of acetic acid was found in the PF_C dough (20.3 ± 1.0 mg/g dw). Concentration of acetic acid in the control dough (12.8 ± 3.5 mg/g dw) was significantly (*p* < 0.05) higher than in the CO_C (3.6 ± 0.6 mg/g dw) and the CO_U dough (1.8 ± 0.1 mg/g dw). On day 3, the highest concentration of acetic acid was found in the PF_C dough (40.3 ± 1.6 mg/g dw) and the lowest concentration was found in the CO_U dough (3.1 ± 0.6 mg/g dw).

Propionic acid was not detected in the control dough throughout the fermentation. In other doughs, the level of propionic acid ranged from 0.9 to 1.7 mg/g dw on day 1. On day 3, the CO_U dough had the lowest concentration of propionic acid (1.4 ± 0.1 mg/g dw) and the level of propionic acid in the other two doughs had no significant (*p* > 0.05) difference and ranged from 7.9 to 9.3 mg/g dw.

### Monosaccharides in Bran Doughs

**FIGURE 2:**
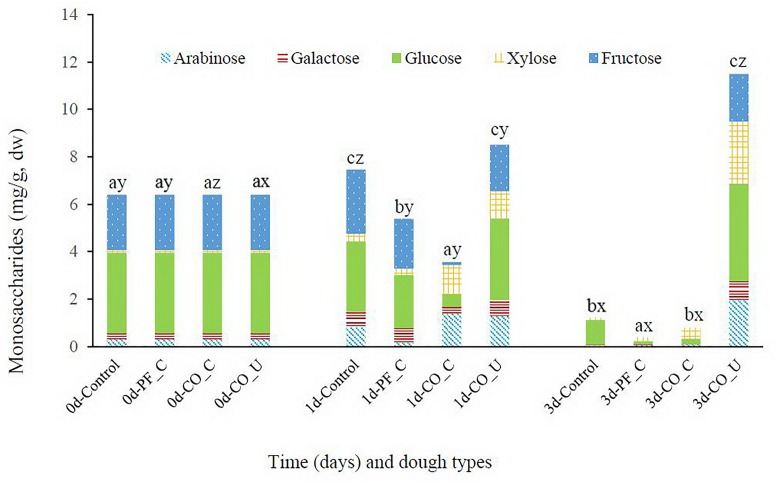
Concentration (mg/g, dry weight) of arabinose, galactose, glucose, xylose, and fructose during fermentation. Values are means and standard deviations of three replicates. Control, spontaneously fermented bran with pH control; PF_C, bran fermented with *P. freudenreichii* monoculture and pH control; CO_C, bran fermented with *P. freudenreichii/L. brevis* co-culture with pH control; CO_U, bran fermented with *P. freudenreichii/L. brevis* co-culture without pH control. Values of total monosaccharide concentration from the same day bearing different superscripts (a–c) are significantly different (*p* < 0.05). Values of total monosaccharide concentration from the same dough type bearing different superscripts (x–z) are significantly different (*p* < 0.05).

On day 0, the main monosaccharides in wheat bran doughs were glucose (3.4 mg/g dw) and fructose (2.3 mg/g dw) while xylose, galactose and arabinose were present at levels ranging from 0.1 to 0.3 mg/g dw (Figure 2). In the control dough, concentration of galactose, xylose and arabinose increased during the first day and the sum of monosaccharides increased from ca. 6.4 mg/g dw to ca. 7.5 mg/g dw. However, only ca. 1.2 mg/g dw of monosaccharides was detected in the control dough on day 3. Content of monosaccharides in the PF_C dough was ca. 5.4 mg/g dw and was mainly composed by glucose and fructose on day 1, while in the CO_C dough there was ca. 3.6 mg/g dw of monosaccharides on day 1, mostly composed of xylose and arabinose. On day 3, there were ca. 0.4 mg/g dw and ca. 0.8 mg/g dw of monosaccharides in the PF_C dough and the CO_C dough, respectively. In the CO_U dough, the concentration of all monosaccharides, except fructose, increased from day 0 to day 3 and reached a sum of ca. 11.5 mg/g dw at the end of fermentation.

### Vitamin B12 in Bran Doughs

**FIGURE 3:**
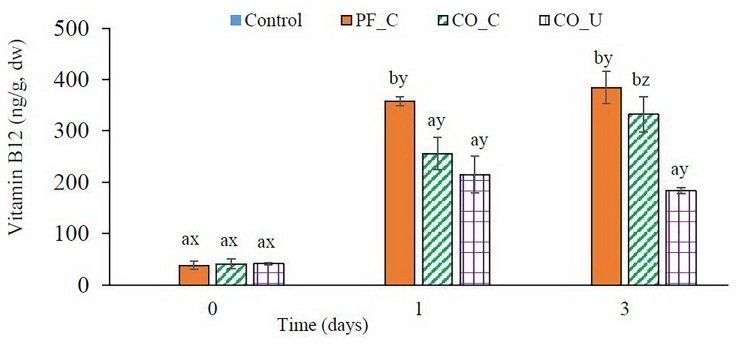
Concentration (ng/g, dry weight) of vitamin B12 during fermentation. Values are means and standard deviation from three replicates. Control, spontaneously fermented bran with pH control; PF_C, bran fermented with *P. freudenreichii* monoculture and pH control; CO_C, bran fermented with *P. freudenreichii/L. brevis* co-culture with pH control; CO_U, bran fermented with *P. freudenreichii/L. brevis* co-culture without pH control. Values from the same day bearing different superscripts (a,b) are significantly different (*p* < 0.05). Values from the same dough type bearing different superscripts (x–z) are significantly different (*p* < 0.05).

In the control dough, no vitamin B12 was detected during fermentation (Figure 3). In other doughs, ca. 40 ng/g dw of vitamin B12 were found on day 0 from the *P. freudenreichii* inoculum. On day 1, the highest content of vitamin B12 was found in the PF_C dough (357 ± 9 ng/g dw). In the CO_C dough and the CO_U dough, vitamin B12 concentration were 255 ± 31 and 214 ± 35 ng/g dw on day 1, respectively. From day 1 to day 3, there was no significant (*p* > 0.05) increase of vitamin B12 concentration in the PF_C dough and the CO_U dough. In the CO_C dough, concentration of vitamin B12 increased to 332 ± 34 ng/g dw on day 3, which was on the same level as in the PF_C dough.

### Riboflavin in Bran Doughs

**FIGURE 4:**
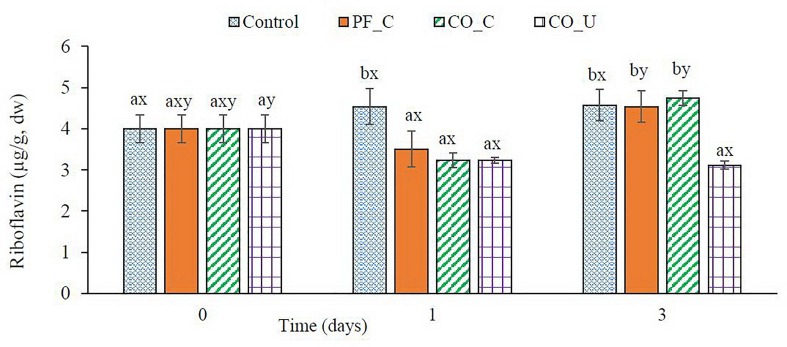
Concentration (μg/g, dry weight) of riboflavin during fermentation. Values are means and standard deviation from three replicates. Control, spontaneously fermented bran with pH control; PF_C, bran fermented with *P. freudenreichii* monoculture and pH control; CO_C, bran fermented with *P. freudenreichii/L. brevis* co-culture with pH control; CO_U, bran fermented with *P. freudenreichii/L. brevis* co-culture without pH control. Values from the same day bearing different superscripts (a,b) are significantly different (*p* < 0.05). Values from the same dough type bearing different superscripts (x,y) are significantly different (*p* < 0.05).

Before fermentation, wheat bran doughs contained ca. 4.0 μg/g dw of riboflavin (Figure 4). In the control dough, concentration of riboflavin had no significant (*p* > 0.05) change during fermentation. In other doughs, riboflavin concentrations were significantly (*p* < 0.05) lower on day 1 varying from ca. 3.2 μg/g dw to ca. 3.5 μg/g dw. From day 1 to day 3, concentration of riboflavin increased to ca. 4.6 μg/g dw in the PF_C dough and the CO_C dough while it remained stable in the CO_U dough.

## Discussion

In the present study, non-sterile wheat bran was used for *in situ* fortification of vitamin B12 by co-fermentation of *P. freudenreichii* DSM 20271 and *L. brevis* ATCC 14869 in controlled conditions. This strain of *P. freudenreichii* was found to be a promising vitamin B12 producer in our previous study (Xie et al., 2018). A pre-screening phase was conducted to select the best performing starter for co-fermentation with *P. freudenreichii* in wheat bran to reduce the growth of *Enterobacteriaceae*, and improve the microbial safety of fermented wheat bran. *L. brevis* ATCC 14869 was selected as it exhibited the strongest antagonistic activity. The adaptability and performance of different strains of *L. brevis* in bran has been shown previously, including the positive effect on the quality of bread containing fermented wheat bran (Coda et al., 2014; Valerio et al., 2014; Messia et al., 2016).

### Microbiological Characteristics of Fermentation

The microbiota of the wheat bran doughs was composed of endogenous microorganisms and the added microbial inocula. In the doughs with starters, the cell density of *P. freudenreichii* was ca. 8.7 log CFU/g before fermentation while the cell density of LAB was ca. 6.3 log CFU/g in the doughs with addition of *L. brevis* and ca. 3.0 log CFU/g in doughs without *L. brevis* inoculation. However, either endogenous or inoculated LAB outnumbered the cell density of *P. freudenreichii* and dominated fermentation already after 1 day of fermentation. Yeasts are a group of microorganisms commonly found in sourdough, co-existing with LAB and the LAB:yeast ratio has been shown to be generally 100:1 during traditional wheat sourdough fermentation (De Vuyst and Neysens, 2005). Similarly to our previous results (Xie et al., 2018), in the present study, LAB:yeast ratio in the control dough was about 10000:1 during fermentation. Meanwhile, addition of *L. brevis* inhibited the growth of yeasts on day 1. However, the cell density of yeasts increased to ca. 5.3 log CFU/g on day 3 in the CO_C dough but remained stable in the CO_U dough suggesting that inhibition of *L. brevis* on yeasts may be due to the decrease of pH.

Native wheat bran also contained some undesirable potential pathogens, such as *Enterobacteriaceae* and *B. cereus*. *Enterobacteriaceae* is a large family of Gram negative bacteria and some members among this group are able to cause infections of the human gastrointestinal tract or might produce various endotoxins (Singh et al., 2015). *B. cereus* is a pathogenic foodborne species commonly existing in plant-origin products such as bread, rice and vegetables (Rosenquist et al., 2005). By producing heat-stable toxins in food, *B. cereus* can cause mild to severe nausea, vomiting and diarrheal illness in humans (Bottone, 2010). Therefore, controlling the growth of these bacteria during bran fermentation is very important for the safety of bran derived food.

Lactic acid bacteria can inhibit the growth of pathogens by production of acids and antimicrobial compounds as well as by competitive exclusion (Kostrzynska and Bachand, 2006). However, outgrowth of *Enterobacteriaceae* (ca. 6.0 log CFU/g on day 1) in the control dough showed that spontaneous fermentation with endogenous LAB was not effective in controlling the cell propagation of *Enterobacteriaceae.* This may be due to the low initial level of endogenous LAB (ca. 3.0 log CFU/g) and pH control condition during fermentation which may diminish the inhibitory effect of produced acids on potential pathogens. Inoculum with *P. freudenreichii* monoculture did not show inhibitory effect on *Enterobacteriaceae* growth either, and under the pH control conditions used here, only the additional starter culture provided a promising inhibition on *Enterobacteriaceae*.

The pre-screening revealed that all the LAB cultures tested could, to a varying extent, inhibit the growth of *Enterobacteriaceae* while *L. brevis* showed the strongest inhibition among them. During co-fermentation with *L. brevis*, the cell density of *Enterobacteriaceae* started to decrease on day 1 irrespective of whether pH was controlled or not. This inhibitory effect of *L. brevis* in the early stage can reduce the microbial risk e.g., potential production of endotoxins during fermentation and increase the overall quality of wheat bran. Moreover, the lower cell density of *Enterobacteriaceae* in CO_U dough (2.8 ± 0.1 log CFU/g) than in CO_C dough (3.4 ± 0.4 log CFU/g) on day 3 showed that the lower pH (3.7 vs. 5.0) can enhance the inhibition of *L. brevis* on *Enterobacteriaceae*. Although in this study the dominance of *L brevis* ATCC 14869 was not confirmed, the high level of inoculum (ca. 6.0 log CFU/g) compared to the endogenous LAB of wheat bran (ca. 3.0 log CFU/g), and the significant (*p* < 0.05) difference of lactic acid (97 vs. 204 ug/g dw) and acetic acid (20 vs. 4 ug/g dw) content between the PF_C dough and CO_C dough suggest that the starter culture was able to steer the fermentation process.

### Utilization of Carbohydrates and Production of Acids

Wheat bran contains various endogenous and microbial enzymes which can result in the release of various monosaccharides from complex carbohydrates during fermentation (Apprich et al., 2014; Immerzeel et al., 2014). Additionally, both inoculated and endogenous microorganisms in bran doughs also consumed monosaccharides to produce acids and other metabolites. In the control dough, endogenous LAB brought intensive acidification. However, from day 0 to day 1, the level of monosaccharides in the control dough increased as a result of liberation of xylose and arabinose by hydrolysis of arabinoxylan, which comprises 10.9 to 26.0% of wheat bran (Onipe et al., 2015).

*Propionibacterium freudenreichii* prefers lactic acid as the carbon source during fermentation and produces propionic acid and acetic acid as the main metabolites (Lee et al., 1974). Addition of *P. freudenreichii* had no effect on the cell density of endogenous LAB but resulted in a faster utilization of monosaccharides and higher production of acids. When *L. brevis* ATCC 14869 was added (CO_C), higher level of lactic acid and lower level of acetic acid were found compared to the dough containing only *P. freudenreichii* (PF_C) and the spontaneously fermented dough on day 1. It was previously observed that co-fermentation of glucose and other carbon sources is a typical feature of *L. brevis* ATCC 14869, in which a less rigorous hierarchical consumption of carbohydrates occurs. Additionally, simultaneous fermentation of glucose and fructose resulted in lactic acid and ethanol as the main products (Kim et al., 2009). After day 1, the level of acetic acid in the CO_C dough increased drastically, likely because of *L. brevis* ATCC 14869 started to use xylose and arabinose after fructose and glucose were almost depleted. In fact, the addition of *L. brevis* largely increased the hydrolysis of arabinoxylan during the first day of fermentation. From day 1 to day 3, microorganisms in doughs with pH control still utilized monosaccharides and continued acid production. However, in the dough without pH control, no further acid production was observed after day 1 suggesting that the low pH reached might have inhibited the metabolic activity of the microorganisms. For example, PAB cannot produce acids when pH is lower than 4.5 (Piwowarek et al., 2018). On the other hand, contents of monosaccharides increased throughout the fermentation because some monosaccharide-releasing enzymes, such as xylanolytic enzymes, may still be active in this pH condition (Bajpai, 2014).

### Production of Vitamin B12

The fact that vitamin B12 was not found in the control dough confirmed that vitamin B12 was only synthesized by the inoculated *P. freudenreichii*. In previous studies, the possibility to fortify plant-based substrates with vitamin B12 by *P. freudenreichii* fermentation was shown. For instance, it was found that 9 ng/g to 37 ng/g fresh weight (41 ng/g to 200 ng/g dry weight) of vitamin B12 were produced by fermentation of *P. freudenreichii* in autoclaved aqueous barley and wheat aleurone matrices (Chamlagain et al., 2017). Moreover, an increase of vitamin B12 content (up to 9.7 ng/g fresh weight) in lupin tempeh by co-fermentation of *Rhizopus oryzae* and *P. freudenreichii* was also reported (Wolkers-Rooijackers et al., 2018). In our former study, ca. 155 ng/g dw of vitamin B12 was produced in wheat bran dough after a 7-day fermentation by *P. freudenreichii* with a final cell density at ca. 9.2 log CFU/g (Xie et al., 2018). In the present study, ca. 200 ng/g dw of vitamin B12 was produced in the CO_U dough after only 1 day of fermentation by *P. freudenreichii* with a cell density at ca. 8.5 log CFU/g. Considering that cobalt is one of the limiting factors for vitamin B12 production by *P. freudenreichii* during fermentation (Hugenschmidt et al., 2011; Deptula et al., 2017; Xie et al., 2018), the higher production of vitamin B12 in the present study was probably due to the higher cobalt content in the wheat bran used here than in the one used in the previous study (0.27 vs. 0.1 μg/g dw; data not shown). In addition, according to Quesada-Chanto et al. (1994), the production of vitamin B12 by *P. freudenreichii* was strongly depending also on pH level. The optimal pH for the production was around 6.5. The higher concentration of vitamin B12 in the PF_C dough than in the CO_C dough on day 1 could be a result of slower acidification in PF_C dough. Moreover, from day 1 to day 3, the concentration of vitamin B12 increased continuously in the CO_C dough, which was not observed in the CO_U dough, implicating that *P. freudenreichii* was still able to produce vitamin B12 at pH 5.0. However, no further increase of vitamin B12 content was observed in the PF_C dough from day 1 to day 3, possibly due to the depletion of available cobalt in the dough.

### Synthesizing Lower Ligand of Vitamin B12 From Riboflavin

Since *P. freudenreichii* mainly produces the active form of vitamin B12 with a 5, 6-dimethylbenzimidazole (DMBI) as the lower ligand (Deptula et al., 2015) and no DMBI were added during fermentation, all the DMBI in the synthesized vitamin B12 was from *de novo* biosynthesis. Riboflavin has been found to be the precursor for the *de novo* biosynthesis of DMBI in *P. freudenreichii* in the presence of oxygen (Hollriegl et al., 1982). After day 1, the content of riboflavin in doughs with vitamin B12 production was significantly (*p* < 0.05) lower than in the control dough confirming that *P. freudenreichii* synthesized DMBI from riboflavin. The content of riboflavin in the PF_C dough and the CO_C dough increased to the same level as in the control dough on day 3 because riboflavin can also be synthesized by *P. freudenreichii* and various LAB species commonly existing in wheat based sourdough microflora (Burgess et al., 2006; Capozzi et al., 2011; Russo et al., 2014).

## Conclusion

This work demonstrated that *P. freudenreichii* can produce nutritionally relevant amount of vitamin B12 in wheat bran during co-fermentation with *L. brevis* and that the vitamin B12 production can be markedly enhanced by maintaining the medium pH above 5. Meanwhile, addition of *L. brevis* with *P. freudenreichii* can effectively inhibit the growth of total *Enterobacteriaceae* and *B. cereus* to ensure the safety of fermentation when pH was controlled around 5. Therefore, wheat bran fermented with *P. freudenreichii* and *L. brevis* can be a promising alternative to produce vitamin B12 enriched ingredient for various food products. These applications could increase the use of wheat bran, thus reducing cereal waste streams and contributing to a more resilient food chain.

## Data Availability

The raw data supporting the conclusions of this manuscript will be made available by the authors, without undue reservation, to any qualified researcher.

## Author Contributions

CX performed the experiments and drafted the manuscript. RC, BC, PV, VP, and KK conceived the experiments and reviewed the manuscript.

## Conflict of Interest Statement

The authors declare that the research was conducted in the absence of any commercial or financial relationships that could be construed as a potential conflict of interest.
