# Supplementary material for: Co-fermentation of Propionibacterium freudenreichii and Lactobacillus brevis in Wheat Bran for in situ Production of Vitamin B12
Source: Front Microbiol. 2019 Jul 5;10:1541. doi: 10.3389/fmicb.2019.01541 (PMC6624789; doi:10.3389/fmicb.2019.01541)
Supplement: Supplementary file 1 [file Data_Sheet_1.docx]

Supplementary Material

**Screening of co-culture strains**

The bran was supplied by Lantmännen Cerealia AB (Malmö, Sweden), contained 14 g protein, 65 g carbohydrates (54 g fibers), 7.1 g ash and 6 g lipids per 100 g.

**Table S 1. Sample codes and strains**

| **Codes** | **Strains** |  |
| --- | --- | --- |
| 1 | *P.* *freudenreichii* ^a^ |  |
| 2 | *P.* *freudenreichii*  ^a^ + *Saccharomyces cerevisiae* H10 ^b^ |  |
| 3 | *P.* *freudenreichii*  ^a^ + *Lactobacillus reuteri* DSM 20016 ^a^ |  |
| 4 | *P.* *freudenreichii*  ^a^ + *Leuconostoc pseudomesenteroides* DSM 20193 ^a^ |  |
| 5 | *P.* *freudenreichii*  ^a^ + *Lactobacillus delcrueckii* ATCC 8000 ^c^ |  |
| 6 | *P.* *freudenreichii*  ^a^ + *Weissella* *confusa* F74 ^d^ |  |
| 7 | *P.* *freudenreichii*  ^a^ + *Leuconostoc*  *mesenteroides*  I21 ^d^ |  |
| 8 | *P.* *freudenreichii*  ^a^ + *Weissella* *confusa* DSM 20194 ^a^ |  |
| 9 | *P.* *freudenreichii*  ^a^ + *Lactobacillus brevis* ATCC 14869 ^c^ |  |

^a^ The strain was obtained from DSMZ (Deutsche Sammlung von Mikroorganismen und Zellkulturen, Germany)

^b^ The strain was obtained from HAMBI culture collection (University of Helsinki, Faculty of Agriculture and forestry, Division of Microbiology and Biotechnology)

^c^ The strain was obtained from ATCC (American Type Culture Collection, USA)

^d^ The strain was from our own culture collection

**Table S 2. PH profile during fermentation**

| **Codes*** | | **0 h** | **12 h** | **24 h** | **36 h** | **48 h** | **60 h** | **72 h** |  |
| --- | --- | --- | --- | --- | --- | --- | --- | --- | --- |
| 1 | | 6.6 | 6.2 | 4.7 | 4.7 | 5.6 | 5.3 | 4.9 |  |
| 2 | | 6.6 | 5.9 | 5.7 | 5.5 | 5.4 | 5.2 | 4.8 |  |
| 3 | | 6.6 | 5.3 | 4.2 | 6.0 | 5.6 | 5.3 | 4.9 |  |
| 4 | | 6.6 | 6.0 | 4.2 | 5.3 | 5.6 | 5.2 | 4.9 |  |
| 5 | | 6.6 | 5.4 | 4.3 | 5.9 | 5.5 | 5.3 | 4.9 |  |
| 6 | 6.6 | | 5.4 | 4.2 | 5.8 | 5.6 | 5.3 | 4.9 |  |
| 7 | | 6.6 | 5.7 | 4.2 | 5.4 | 6.0 | 5.7 | 4.9 |  |
| 8 | | 6.6 | 5.5 | 4.2 | 5.6 | 5.6 | 5.5 | 5.0 |  |
| 9 | | 6.6 | 4.8 | 4.2 | 4.6 | 5.9 | 5.5 | 5.2 |  |

* Details about the sample codes can be found in Table S1.

**Table S 3. Cell count (log CFU/g) of *Enterobacteriaceae* during fermentation.**

* Details about the sample codes can be found in Table S1.

| **Codes*** | **Day 0** | **Day 1** | **Day 3** |  |
| --- | --- | --- | --- | --- |
| 1 | 3.7 ± 0.1 | 5.9 ± 0.4 | 6.2 ± 0.3 |  |
| 2 | 3.8 ± 0.0 | 5.0 ± 0.1 | 5.3 ± 0.2 |  |
| 3 | 3.8 ± 0.1 | 3.2 ± 0.2 | 3.6 ± 0.2 |  |
| 4 | 3.8 ± 0.1 | 3.4 ± 0.1 | 4.0 ± 0.3 |  |
| 5 | 3.7 ± 0.0 | 2.4 ± 0.0 | 4.0 ± 0.1 |  |
| 6 | 3.8 ± 0.1 | 2.7 ± 0.1 | 3.2 ± 0.4 |  |
| 7 | 3.8 ± 0.2 | 3.1 ± 0.2 | 3.9 ± 0.1 |  |
| 8 | 3.8 ± 0.1 | 3.0 ± 0.2 | 3.6 ± 0.2 |  |
| 9 | 3.8 ± 0.2 | 2.4 ± 0.2 | 3.2 ± 0.2 |  |

**Table S 4. Cell count (log CFU/g) of *Propionibacterium* *freudenreichii* during fermentation.**

| **Codes*** | **Day 0** | **Day 1** | **Day 3** |  |
| --- | --- | --- | --- | --- |
| 1 | 8.6 ± 0.0 | 9.3 ± 0.2 | 9.2 ± 0.2 |  |
| 2 | 8.6 ± 0.1 | 9.4 ± 0.2 | 9.4 ± 0.2 |  |
| 3 | 8.6 ± 0.1 | 9.3 ± 0.1 | 9.3 ± 0.1 |  |
| 4 | 8.7 ± 0.2 | 9.4 ± 0.2 | 9.3 ± 0.2 |  |
| 5 | 8.6 ± 0.1 | 9.0 ± 0.1 | 9.1 ± 0.1 |  |
| 6 | 8.6 ± 0.1 | 8.9 ± 0.2 | 9.0 ± 0.2 |  |
| 7 | 8.6 ± 0.0 | 9.1 ± 0.2 | 9.0 ± 0.1 |  |
| 8 | 8.7 ± 0.1 | 9.4 ± 0.1 | 9.4 ± 0.2 |  |
| 9 | 8.7 ± 0.1 | 9.1 ± 0.1 | 8.9 ± 0.2 |  |

* Details about the sample codes can be found in Table S1.
